# Addition of heart score to high-sensitivity troponin T versus conventional troponin T in risk stratification of patients with chest pain at the coronary emergency rooms

**DOI:** 10.1007/s12471-014-0600-6

**Published:** 2014-10-08

**Authors:** M. N. I. Willems, D. J. van de Wijngaart, H. Bergman, A. Adiyaman, D. Telting, F. F. Willems

**Affiliations:** 1 Department of Cardiology, Wagnerlaan 55, 6800 TA Arnhem, the Netherlands; 2 Laboratory of Clinical Chemistry and Haematology, Rijnstate Hospital, Wagnerlaan 55, 6800 TA Arnhem, the Netherlands; 3 Department of Cardiology, Rijnstate Hospital, Wagnerlaan 55, 6800 TA Arnhem, the Netherlands; 4 Department of Cardiology, Isala Klinieken, Groot Wezenland 20, PO box 10500, 8000 GM Zwolle, the Netherlands; 5 Sint Anna Zorggroep, Klinisch Laboratorium, Bogardeind 2, 5664 EH Geldrop, Postbus 90, 5660 AB Geldrop, the Netherlands

**Keywords:** High-sensitivity troponin T, Cardiovascular risk, Cardiac emergency room

## Abstract

Patients with chest pain have a large impact on available resources in coronary emergency rooms (CER). Clinical judgement, ECG, risk scores and biomarkers guide in risk stratification. We investigated if high-sensitivity troponin T (HsT) and the HEART Score could contribute to risk stratification at the CER. All patients with chest pain, without elevated conventional troponin levels at presentation, were included. HsT levels were determined at admission (T1), at 4–6 h (T2) and 8–10 h after symptom onset (T3). The HEART Score was calculated as risk score for the occurrence of a major adverse cardiac event (MACE). Thirty days after discharge, occurrence of MACE was registered. Eighty-nine patients were included (overall mean age 61 years (range 20–90)). At presentation, 68 patients (76 %) had a HsT below cut-off value of 14 ng/l (mean HEART Score 3.7, range 1–9). Thirty-one of these 68 patients had a HEART Score between 1–3, no MACE occurred in this group. For 3 patients (4 %) HsT levels increased above 14 ng/l. These 3 patients had a HEART Score between 4–6. The majority of patients with chest pain can be safely discharged within 4–6 h after onset of symptoms using HsT and the HEART Score. In contrast, patients with initially normal HsT but a high HEART Score need longer follow-up and repeat HsT determination.

## Introduction

An increasing number of patients with chest pain are referred to the casualty department or the coronary emergency room (CER) to prove or exclude a potentially serious disease such as an acute coronary syndrome. The challenge lies in rapidly identifying patients who can safely be discharged from the CER. In addition, it is necessary to identify patients who need an aggressive treatment, as part of an acute coronary syndrome (ACS), and to take appropriate action as soon as possible.

The diagnosis of chest pain is made for 47 % of all patients who are admitted to the cardiology department [1]. These numbers include patients with ACS. ACS is not responsible for symptoms in 40 % of patients presenting with chest pain in the CER. The average period of stay of these patients at the CER in our hospital is currently 11 h. Given the increasing pressure on the capacity of beds, a shorter length of stay is warranted. The use of biomarkers in addition to risk scores can be helpful in optimising the discharge policy in the CER, through which a shorter duration of stay might be achieved.

This study was designed to investigate and evaluate the opportunities for early discharge created by the availability of new and more sensitive biomarkers such as high-sensitivity troponin T assays (HsT) [2–4]. Our study focuses on the extent to which the HsT in combination with a risk score contributes to the optimisation of earlier decision-making and discharge policy (4–6 h) in the CER.

## Methods

This study was designed as a prospective cohort study at the CER of the Rijnstate Hospital in Arnhem in the period from February 2011 to July 2011. The study was approved by the local ethics committee and 131 patients initially participated. The clinical cardiologist and the patients were blinded for HsT levels.

For this study, all patients presenting to the CER with chest pain were eligible. Patients could be enrolled if the symptoms had been present for less than 6 h. Excluded were patients with an indication for percutaneous coronary intervention (PCI), as were patients with ST-elevation myocardial infarction (STEMI) or non-ST-elevation myocardial infarction (NSTEMI; typical angina with elevated conventional troponin T) diagnosed at admission. In addition, patients with chronically elevated conventional troponin T were excluded. Finally, patients who had been treated for a cardiovascular complication (e.g. heart failure, unstable angina or myocardial infarction) in the past month were also excluded. All patients were diagnosed and treated based on conventional troponin T. According to the discretion of the cardiologist, non-invasive ischaemia detection could be performed after discharge, during follow-up. Myocardial infarction was defined as a rise and/or fall of conventional troponin with at least 1 value above 30 ng/l [2].

Patients presenting to the CER underwent an initial clinical evaluation and, as well as the routine laboratory tests including determination of the conventional troponin T, an additional sample was taken to determine the HsT (T1). This was repeated at 4–6 h (T2) and 8–10 h after symptom onset (T3). If patients presented to the CER within the window of T2, only the T2 sample was taken. As with conventional troponin T, HsT was measured on the E170 module of the Modular Immunoassay (Roche Diagnostics). We used 14 ng/l as a cut-off value for HsT, above which results were considered to be ‘increased’. For the conventional troponin T assay, this value was set at 30 ng/l.

The 99^th^ percentile for conventional troponin is 10 ng/l. The 10 % coefficient of variation is set at 30 ng/l. The limit of detection for conventional troponin is 10 ng/l. The 99^th^ percentile for the HsT is 14 ng/l. The coefficient of the variation of 10 % is set at 13 ng/l. The limit of detection for the HsT is 5 ng/l.

The conventional troponin T does not meet the requirement that the coefficient of variation at the 99^th^ percentile limit is below 10 % [5].

In addition, the HEART Score was calculated using the conventional troponin T value. The HEART Score is a scoring system for patients presenting to the CER with chest pain [6, 7]. By assigning zero, one or two points to an atypical patient history, ECG abnormalities, the patient’s age, any risk factors present, and elevated troponin, patients receive a score on a scale of 0–10. Patients who score 0-3 points have <1 % chance of developing a cardiac event, those scoring 4–6 points have a 12 % chance, while those patients with a score of ≥7 have on average a 65 % chance of having a myocardial infarction, percutaneous coronary intervention or coronary artery bypass graft, or death within six weeks after presentation (MACE) [6, 7]. Thirty days after discharge, the presence of MACE during follow-up was registered retrospectively by checking electronic patient files and personal telephone communication with the patient, with additional information from the general practitioner, when needed.

All statistical analyses were performed with SPSS 18.0.

## Results

For this study, 131 patients were eligible for enrolment. Forty-two patients were excluded on basis of the exclusion criteria. Finally, 89 patients were included in this study, 52 males and 37 females with an overall mean age of 61 years (range 20–90 years). Admission time to the CER varied considerably, ranging from 30 min to 6 h after symptom onset. Baseline characteristics are shown in Table 1. Table 2 shows the range and means of the values of HsT and conventional troponin T assays measured at the different time points. Table 3 shows the results of the combination of HsT and the HEART Score at both T2 and T3, with the incidence of MACE during follow-up. Patients with HsT < 14 ng/l at T2 and T3, could have MACE during follow-up, whereas no patients with a HEART Score ≤ 3 had MACE during follow-up.

**Table 1:** Baseline characteristics

N = 89	
Gender	52 male, 37 female
Age	Mean 61 year (range 20–90)
Duration of symptoms	Mean 196 min (range 30–360)
HEART Score (using cTnT)	Mean 4.15 (range 1–8)
History of vascular disease	39 (44 %)
Diabetes mellitus	17 (19 %)
Smoking	25 (28 %)
Hypertension	29 (32 %)
Family history of vascular disease	30 (34 %)
Hypercholesterolaemia	32 (36 %)

**Table 2:** Descriptive values of the conventional Troponin T and the HsT assays

Conventional Troponin (ng/l)	N	Minimum	Maximum	Mean	SD
T1 (Entry)	89	16.0	30.0	29.6	1.9
T2 (4 h – 6 h)	89	11.0	333.0	32.9	32.3
T3 (8 h – 10 h)	89	13.0	661.0	41.5	75.0
HsT (ng/l)	N	Minimum	Maximum	Mean	SD
T1 (Entry)	89	3.0	71.9	10.4	12.4
T2 (4 h – 6 h)	89	3.0	347.0	14.7	37.9
T3 (8 h – 10 h)	89	3.0	675.4	24.4	83.8

**Table 3:** Combining HsT with HEART Score at T2 and T3 in order to calculate the risk for MACE

HsT (T2)	N	HEART Score	MACE	No MACE
≤ 14 ng/l	31	1–3	0	31
(*N* = 68)	34	4–6	3	31
	3	7–10	0	3
≥ 14 ng/l	0	1–3	0	0
(*N* = 21)	13	4–6	3	10
	8	7–10	3	5
HsT (T3)	N	HEART Score	MACE	No MACE
≤ 14 ng/l	31	1–3	0	31
(*N* = 65)	31	4–6	2	29
	3	7–10	0	3
≥ 14 ng/l	0	1–3	0	0
(*N* = 24)	16	4–6	4	12
	8	7–10	3	5

Sixty-eight patients out of 89 (76 %) presented with HsT values below 14 ng/l. Thirty-one of these patients (46 %) had a HEART Score between 1–3. In this group no MACE occurred within the one-month follow-up period.

Thirty-seven patients (42 %) with HsT levels <14 ng/l at T2 had a HEART Score above 3. In this group, 3 MACE occurred in the one-month follow-up (8 %). These 3 patients all had a PCI because of significant coronary stenosis (angiographically > 70 % and/or fractional flow reserve < 0.8) during 30 days of follow-up.

In 21 patients (24 %) elevated HsT levels were observed at T2. The probability of a cardiac event in this group was substantially higher (28 %). In this group, there were no patients with a low HEART Score.

**Fig 1:**
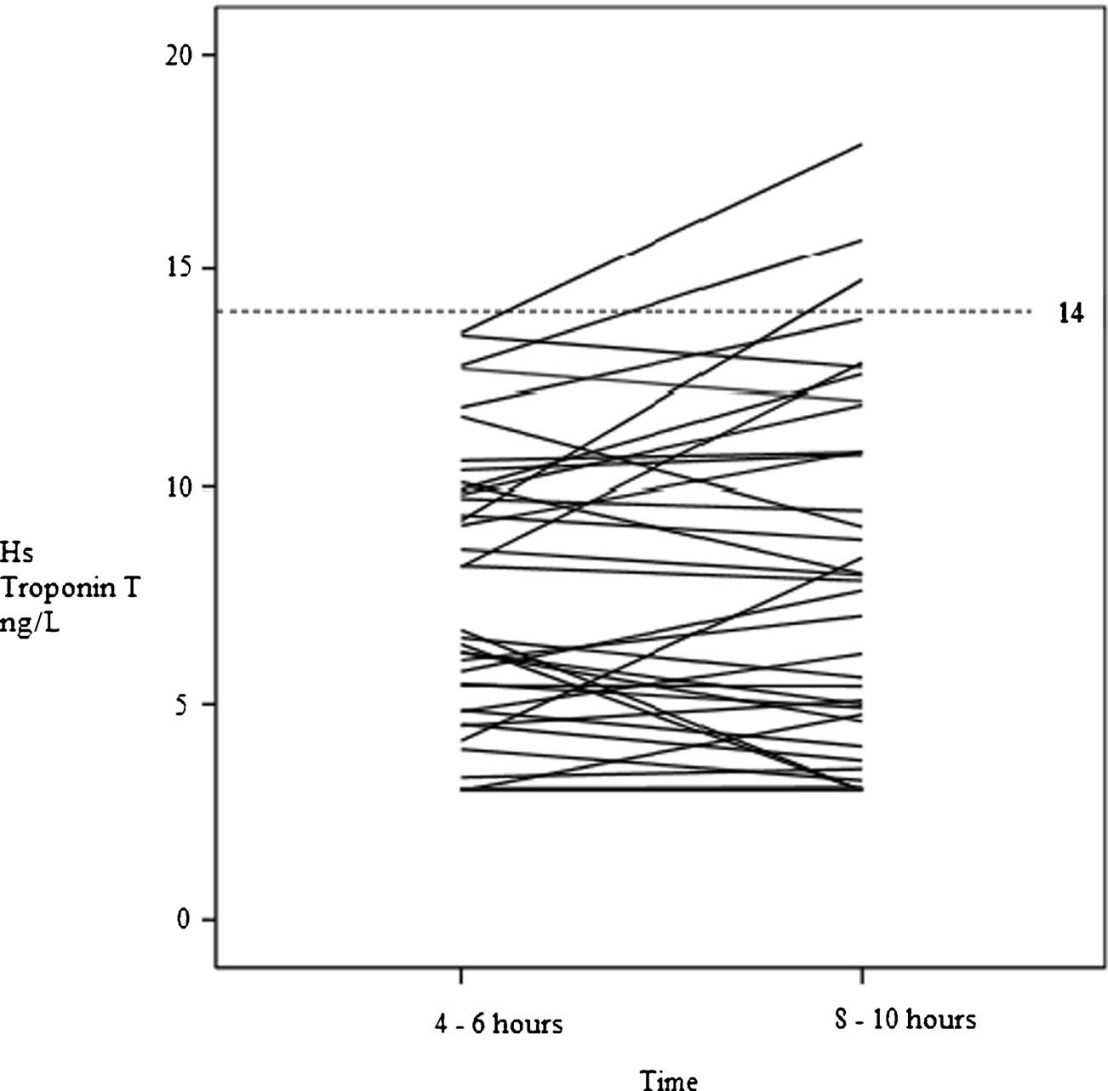
eps Changes in serum high sensitivity troponin T (HsT) in patients with a level ≤ 14 ng/L at 4-6 h after onset of symptoms

In Fig. 1 HsT levels at T2 and T3 are compared. At T2 (4–6 h after onset of symptoms), 68 patients had a HsT value less than 14 ng/l. At T3 (8–10 h after start of symptoms), for 3 patients (4 %) HsT levels increased to levels above the cut-off value of 14 ng/l (average increase in these 3 patients was 30 %). All 3 patients had a moderately increased HEART Score between 4 and 6. Based on their HEART Score, they need longer follow-up and repeat HsT determination. One patient had occurrence of a MACE during follow-up.

## Discussion

We investigated the value of the HEART Score as risk stratification model, with the use of conventional troponin T or HsT in patients with suspected ACS. Our main findings were:

Patients with normal HsT and a HEART Score of ≤ 3 could safely be discharged at T2, as no MACE occurred. With a HEART Score of ≥ 4 or more, further monitoring, serial troponin tests, and/or extra investigation (exercise testing, nuclear imaging, stress echocardiography) may be required, because a small proportion of patients have MACE at follow-up. Patients with elevated HsT at T2 and T3, and/or with a HEART Score ≥ 7 are at high risk of MACE. These patients should be admitted and coronary angiography can be considered.

The HEART Score is a specially validated risk score for patients with chest pain presenting to an emergency department in the Netherlands [6, 7]. Other risk scores such as the GRACE or TIMI risk score are mainly validated in patients with STEMI and NSTEMI and therefore less useful for this category of patients [8, 9]. It should be noted that these risk scores include elevated cardiac markers in their point scoring system. The HEART Score system makes use of conventional troponin, and this score system should be further validated, especially with the current widespread use of HsT in cardiology practice.

In our present study, 3 patients (4 %) with initially normal HsT at T2 showed a rise above the cut-off value of 14 ng/l at T3. The significance of such an increase is at present unclear, as some advocate that a 100 % rise is associated with acute, thrombotic, myocardial infarction. If clinical decision-making had been based on HsT alone, these 3 patients would not be identified as high-risk patients at risk for MACE. By also taking the HEART Score into account, we could identify these patients at T2. This underscores the importance of combining risk scores with laboratory tests.

The use of a risk score as the HEART Score can provide additional information for identifying these patients.

As mentioned earlier, the HEART Score has been validated for conventional troponin T and not for HsT. It is to be expected that by using a high-sensitivity assay in a new HEART Score, HsT levels will more often be elevated as compared with conventional troponin T, and hereby the new risk score could have a higher sensitivity, at the cost of less specificity. Further studies should provide conclusions regarding the discriminative value of this new risk score.

Despite the fact that cardiac troponins are sensitive and are specific biochemical markers of myocardial damage, an increased troponin T level occurs in many diseases in which the heart does not show ischaemia, such as congestive heart failure, pulmonary embolism, renal failure, acute neurological disease, myocarditis or sometimes in apparently healthy persons [10]. A clinical observation period may therefore be required.

Furthermore, the availability of new high-sensitive assays for troponin T may allow the detection of small changes in troponin and this test meets the requirements of the universal definition of myocardial infarction. The universal definition of acute myocardial infarct states that there has to be a typical rise and/or fall of biochemical markers (preferably troponin) with at least 1 value above the 99^th^ percentile reference limit with at least one of the following: ischaemic symptoms, ECG changes, pathological Q waves or imaging evidence of new loss of viable myocardium motion abnormality [2]. The HsT assay does meet the requirement that the coefficient of variation at the 99^th^ percentile limit is below 10 %. The test does not only appear more sensitive, but is able, even 3 h after symptom onset, to differentiate whether there is an acute coronary syndrome or not. Early decision-making is therefore possible [10]. A problem that may arise is the possibility of false-positive results of acute coronary syndrome. However, it is still unclear whether there should be an absolute or relative increase in troponin values. In addition, at this moment it is also unclear how much the troponin values should increase to speak about an acute coronary syndrome. Some advocate that a doubling of the serum HsT within 3 h indicates an acute coronary syndrome whereas others (including the guidelines) state that there has to be a rise and/or fall with at least 1 value above 14 ng/l [11].

Our study is relatively small; therefore, future studies should be aimed at extending these findings by including more patients. Only with evidence from larger study populations, conclusions for clinical decision-making can be drawn. Our present study is therefore mainly hypothesis generating at this moment.

In conclusion, our study suggests that patients with chest pain at the CER, with a negative HsT level together with a low HEART Score, can be safely discharged from the hospital within 4-6 h after onset of symptoms. The HEART score should be further clinically validated with HsT in larger patient cohorts.
